# Using electromagnetic navigation for intraoperative rib fracture localization during rib plating: A case report

**DOI:** 10.1016/j.ijscr.2018.11.029

**Published:** 2018-11-22

**Authors:** Rodrigo Pedraza, Edward Y. Chan, Leonora M. Meisenbach, Min P. Kim

**Affiliations:** a Division of Thoracic Surgery, Department of Surgery, Houston Methodist Hospital, Houston, TX, United States; b Department of Surgery, Weill Cornell Medical College, Houston Methodist Hospital, Houston, TX, United States

**Keywords:** CT, computed tomography, 3D, three-dimensional, EM, electromagnetic, VATS, video-assisted thoracoscopic surgery, Case report, Rib fractures, Rib fixation, Rib plating, Electromagnetic navigation

## Abstract

**Introduction:**

The precise localization of fractured ribs represents one of the primary challenges of surgical rib fixation. Computed tomography (CT) provides the facture’s general location, but it is difficult to use the imaging alone to properly place a surgical incision. We used electromagnetic navigation to identify the exact location of the fracture on the patient’s skin.

**Presentation of case:**

A 64-year-old man fell and suffered multiple left-sided nonunion rib fractures (4^th^ to 9^th^). He was initially treated with a chest tube and analgesia, but he developed chronic pain from the injury. On the CT scan of the chest, the rib fractures were displaced and on exam, the ribs were mobile with reproducible pain and clicks on palpation. We used electromagnetic navigation to determine the fracture’s exact location on the skin during the operation, which aided in the location of the incision. The patient had open reduction and internal fixation of the broken ribs using rib plates. The patient had relief from his chronic pain after the surgery.

**Discussion:**

The localization of the rib fracture on a patient’s skin can be performed using a physical exam, landmarks from the CT scan, or video-assisted thoracic surgery (VATS) procedure. Each of the techniques sacrifices either time or accuracy during the operation. The electromagnetic navigation can provide precise localization of the fracture without sacrificing too much time during the operation.

**Conclusion:**

The use of electromagnetic navigation to identify the fracture on a patient’s skin provides quick and accurate intraoperative localization for surgical rib fixation.

## Highlights

• The precise localization of the fractured rib on the patient’s skin can facilitate rib plating.
• Electromagnetic navigation can identify the precise location of the fracture on the skin.

## 1 Introduction

Rib fractures are most commonly traumatic and are typically treated with the use of analgesics and pulmonary toilet. In an acute setting, surgical rib fixation is typically reserved for patients with flail chest, acute respiratory failure, and failure to wean from mechanical ventilation [1]. Delayed operative fixation has been recommended in patients with chronic symptomatic nonunion rib fractures [2,3].

Preoperative planning requires a computed tomographic (CT) scan with three-dimensional (3D) reconstruction. While the CT scan allows for a 3D conceptualization of the rib fractures’ location, it does not account for cutaneous landmarks [4]. It provides a general idea of the fracture’s location, which leads to larger than absolutely necessary incisions to obtain proper exposure of the broken rib. In order to provide a more accurate location of the fractured rib, thoracoscopic guidance is used to identify the location of the rib fracture [4]. However, there are often dense adhesions involving the lung secondary to the index injury and any subsequent hemothorax, necessitating extensive adhesiolysis to identify the fracture’s exact location. We wanted to determine if there is a better way to identify the rib fracture on patient’s skin.

One of the advances in the field of lung nodule identification has been the development of electromagnetic (EM) navigation bronchoscopy. This technology uses an EM field to create a real time endobronchial road map to the peripheral nodule using a CT scan. First, a CT scan of the chest is performed, and the images are imported into a software that creates a virtual 3D image of the airway and the nodule is identified in the virtual airway. Second, an endoscopy is performed to match the real airway to the virtual airway [5]. Third, the virtual airway is used to guide the catheter to the nodule for a biopsy through the airway. The same concept can be used to locate the nodule through the chest wall using a protocol called SPiN Perc^™^ (Veran Medcial Technologies, St. Louis, MO) [6]. We present a case report of a novel method to precisely identify the location of a rib fracture on the skin using an electromagnetic navigation system with SPiN Perc^™^ protocol.

This work has been reported in line with SCARE criteria [7].

## 2 Case report

A 64-year-old Caucasian man fell in a bathtub approximately 9 months prior to presentation. At the time of the injury, he suffered multiple left-sided nonunion rib fractures (4^th^ to 9^th^) and was treated with a chest tube and analgesia. He had developed chronic pain from the injury to the point in which he was unable to sleep at night and heard a clicking sound every time he took a deep breath. On the CT scan of the chest, the rib fractures were displaced and on exam, the ribs were mobile with reproducible pain and clicks on palpation. The patient was taken to the operating room for an open reduction and internal fixation with MatrixRIB fixation plates (DePuySynthes, West Chester, PA, USA).

**Fig. 1:**
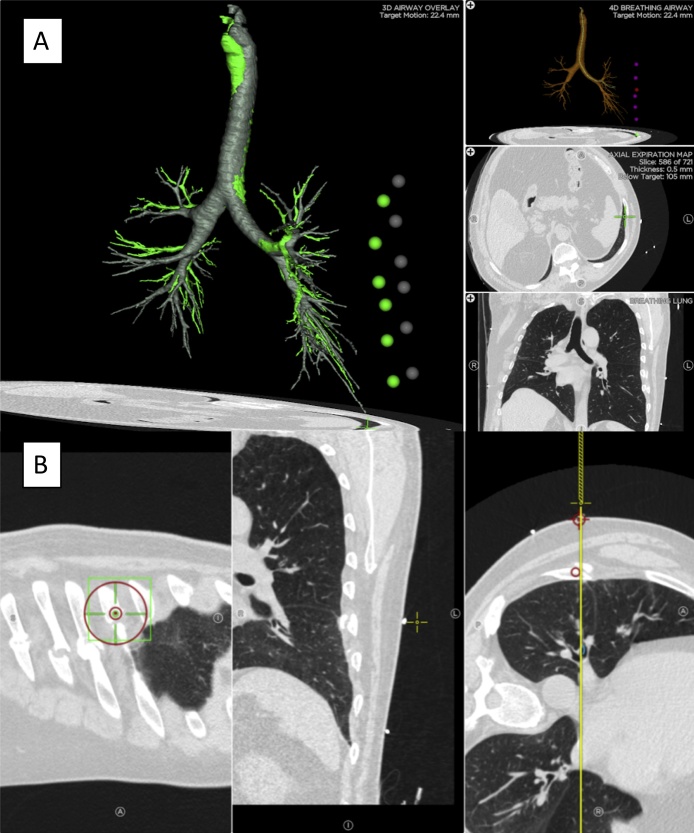
Planning for Localization. (A) Screenshot of the planning software with visualization of all targets during inspiration (green dots) and expiration (grey dots). Each individual rib fracture site is marked as a target prior to viewing this summary screen. There is 2.2 cm displacement of the rib between inspiration and expiration. (B) Screenshot of the planning software for localization of the rib fracture on the skin using the SPiN Perc^™^. An entry site on the skin is placed perpendicular to the site of the rib fracture.

**Fig. 2:**
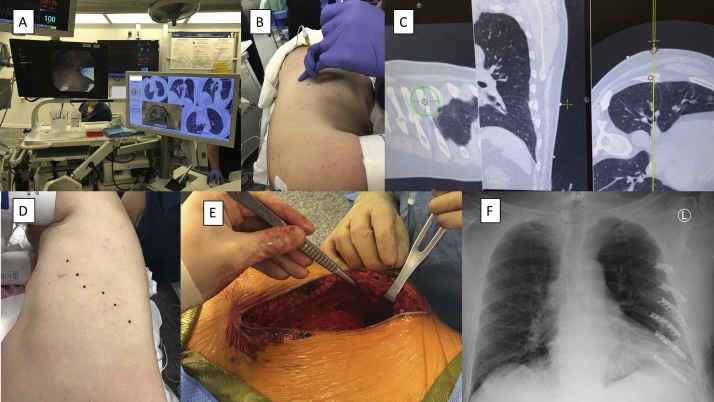
Intra-operative Localization. (A) Photograph of the bronchoscopy screen on the left and the electromagnetic navigation screen on the right correlating the bronchoscopy of carina to software-generated carina to match the computed tomographic (CT) image to the patient. (B–C) Photograph of real time localization using SPiN Perc^™^ software to mark the location of the fracture on the skin. The needle tip (B) is moved to the corresponding spot seen on the software screen (C). (D) Photograph of the skin after localization. (E) Photograph of the needle through the skin accurately locating the rib fracture. (F) Chest X-ray after rib fixation using the MatrixRIB plating system.

The procedure was performed by a board certified thoracic surgeon (MPK). On the day of the operation, the patient had a dynamic CT scan, with one scan taken with maximal inspiration and one scan taken with maximal expiration in the right lateral decubitus position with the left side up. The CT scan was imported into the electromagnetic navigation system software (Veran Medical Technologies Inc, St. Louis, MO). We first marked the rib fractures on the planning software as a target. The software demonstrated 2.2 cm rib fracture displacement between inspiration and expiration (Fig. 1A). Next, we identified the skin that was directly superficial to the site of the fractured ribs using the SPiN Perc^™^ protocol (Fig. 1B). Intraoperatively, a bronchoscopy was performed for electromagnetic calibration (Fig. 2A). This allows the accurate matching of the patient’s anatomy to the virtual anatomy seen on the CT scan. Then, cutaneous sites of all fractures were identified and marked with image guidance (Fig. 2B-C). These markings served as a guide for the incision’s location. After incision, the dissection was continued to the chest wall sparing the latissimus dorsi and serratus anterior. The rib fractures were readily identified with accurate correlation with the cutaneous sites (Fig. 2D). The fractures were freed from the bony callus and were fixed with MatrixRIB plates (DePuySynthes, West Chester, PA, USA) in a conventional fashion (Fig. 2E). A closed suction drain was left in the operative field. The patient recovered well postoperatively and reported immediate improvement in chest wall pain. He was discharged on postoperative day 2 with non-narcotic analgesics.

## 3 Discussion

The current guidelines and recommendations for the management of traumatic rib fractures are based on consensus and/or expert opinion, given the paucity of high-quality research. In general, most traumatic (non-pathologic) rib fractures are managed conservatively and few require surgical intervention, which is more often performed in the acute setting. More recently, however, the long-term functional impact of rib fractures has been investigated [8]. It has been demonstrated that nearly one-third of patients with rib fractures develop chronic pain and nearly half of them have a chronic disability. Moreover, rib fracture fixation for symptomatic nonunion has been shown to be safe, feasible, and potentially beneficial [2]. Patients with nonunion usually complain of chronic pain, shortness of breath, and “clicking” with breathing [3]. Our patient had chronic pain since the trauma as well as a “clicking” sensation with deep breaths that disrupted his daily life and sleep.

While reviewing our patient’s CT scan, it was noted that the fractures were mildly displaced. However, on dynamic imaging, this displacement became more pronounced explaining the persistent symptoms and supporting the indication of surgical fixation. It was also noted that the superior fractures were anterolateral while the inferior fractures were posterolateral, which posed a challenge for surgical planning. We were able to identify all of the fractures in this patient using the software. To our knowledge, there is no limit in terms of the number of rib fractures that can be identified by the software and it can be used for acute and chronic setting. Moreover, it has been suggested that video-assisted thoracoscopic surgical (VATS) guidance may facilitate incision planning [4], but this would require double lumen intubation with possible lysis of adhesions of the lung, and the need for a postoperative thoracostomy tube. Furthermore, in a patient with a large amount of visceral fat, VATS visualization of rib fractures may be challenging. The use of electromagnetic navigation in our patient facilitated placement of the incision with optimal exposure to all rib fractures for fixation.

After a literature review, to our knowledge, this is the first reported case of preoperative rib fracture localization with the use of electromagnetic navigation for incision planning. This technology afforded proper fracture site localization at the skin level, which permitted the creation of a single incision accomplishing access to all fractures for fixation.

## Conflicts of interest

The authors have no conflicts of interest or financial ties in relation to this manuscript.

## Sources of funding

None.

## Ethical approval

The study was approved by the Institutional Review Committee at Houston Methodist Research Institute.

## Consent

The patient provided informed consent for publication.

## Author contribution

Study concept: Rodrigo Pedraza and Min P. Kim

Data collection: Rodrigo Pedraza, Edward Y. Chan, Leonora M. Meisenbach and Min P. Kim

Data analysis or interpretation: Rodrigo Pedraza, Edward Y. Chan, Leonora M. Meisenbach and Min P. Kim

Writing the paper: Rodrigo Pedraza and Min P. Kim

Critical review and final approval: Rodrigo Pedraza, Edward Y. Chan, Leonora M. Meisenbach and Min P. Kim.

## Registration of research studies

No registered.

## Guarantor

Min Kim.

## Provenance and peer review

Not commissioned, externally peer reviewed.

## Disclosure

The authors have no conflicts of interest or financial ties in relation to this manuscript. MPK consults for Veran, Medtronic, Intuitive Surgical, and Olympus. EYC consults for Medtronic, Veran and Olympus.

## References

[1] Marasco S., Saxena P. (2015). Surgical rib fixation - technical aspects. Injury.

[2] de Jong M.B., Houwert R.M., van Heerde S., de Steenwinkel M., Hietbrink F., Leenen L.P.H. (2018). Surgical treatment of rib fracture nonunion: a single center experience. Injury.

[3] Gauger E.M., Hill B.W., Lafferty P.M., Cole P.A. (2015). Outcomes after operative management of symptomatic rib nonunion. J. Orthop. Trauma.

[4] Sarani B., Schulte L., Diaz J.J. (2015). Pitfalls associated with open reduction and internal fixation of fractured ribs. Injury.

[5] Semaan R.W., Lee H.J., Feller-Kopman D., Lerner A.D., Mallow C.M., Thiboutot J. (2016). Same-day computed tomographic chest imaging for pulmonary nodule targeting with electromagnetic navigation bronchoscopy may decrease unnecessary procedures. Ann. Am. Thorac. Soc..

[6] Yarmus L.B., Arias S., Feller-Kopman D., Semaan R., Wang K.P., Frimpong B. (2016). Electromagnetic navigation transthoracic needle aspiration for the diagnosis of pulmonary nodules: a safety and feasibility pilot study. J. Thorac. Dis..

[7] Agha R.A., Fowler A.J., Saeta A., Barai I., Rajmohan S., Orgill D.P. (2016). The SCARE statement: consensus-based surgical case report guidelines. Int. J. Surg..

[8] Gordy S., Fabricant L., Ham B., Mullins R., Mayberry J. (2014). The contribution of rib fractures to chronic pain and disability. Am. J. Surg..

